# Pain and Clinical Presentation: A Cross-Sectional Study of Patients with New-Onset Chronic Pain in Long-COVID-19 Syndrome

**DOI:** 10.3390/ijerph20054049

**Published:** 2023-02-24

**Authors:** Andrés Calvache-Mateo, Laura López-López, Javier Martín-Núñez, Alejandro Heredia-Ciuró, María Granados-Santiago, Araceli Ortiz-Rubio, Marie Carmen Valenza

**Affiliations:** 1 Physiotherapy Department, Faculty of Health Sciences, University of Granada, 18016 Granada, Spain; 2 Nursing Department, Faculty of Health Sciences, University of Granada, 18016 Granada, Spain

**Keywords:** long-COVID-19 syndrome, new-onset pain, pain characteristics

## Abstract

The aim of this study was to evaluate the characteristics of pain (i.e., pain intensity, pain interference, clinical presentation) in Long-COVID-19 patients and compare the location of pain between successfully recovered COVID-19 patients and healthy matched controls. A cross-sectional case-control study was carried out. Long-COVID-19 patients, age- and sex-matched patients with a history of COVID-19 who had successfully recovered, and healthy controls were included. Outcomes included were pain characteristics (Brief Pain Inventory and Short-Form McGill Pain Questionnaire) and clinical presentation (Widespread Pain Index and Euroqol-5 Dimensions 5 Levels Visual Analogue Scale). Sixty-nine patients with Long-COVID-19 syndrome, sixty-six successfully recovered COVID-19 patients, and sixty-seven healthy controls were evaluated. Patients with Long-COVID-19 syndrome showed greater pain intensity and interference. In addition, they showed worse quality of life and greater widespread pain, with the most frequent locations of pain being the neck, legs, and head. In conclusion, patients with Long-COVID-19 syndrome show a high prevalence of pain, characterized by widespread pain of moderate intensity and interference, with the most frequent locations being the neck, legs, and head, significantly affecting the quality of life of these patients.

## 1. Introduction

The COVID-19 pandemic has impacted the lives and health of persons worldwide, with the potential for further effects in the future. The repercussion of this pandemic extends beyond physical illness, with relevant psychosocial consequences significantly impacting all health-related areas [1]. In this context of uncertainty, some of those who have recovered from COVID-19 infection develop persistent or new symptoms lasting weeks or months; this has been called “Long-COVID-19” or “post-COVID-19 syndrome” [2]. This new entity can be continuous or relapsing and remitting in nature [3] and can develop the persistence of one or more symptoms of acute COVID or an appearance of new symptoms.

The most commonly referenced symptoms were fatigue (53%), dyspnea (43%), and pain (27%). In a large study evaluating the reported symptoms of Long-COVID-19 syndrome, 76% of patients reported at least one of a list of 17 symptoms, with the most frequent being fatigue and muscular weakness (63%), sleep difficulties (26%), and anxiety or depression (23%) [4]. However, the reviewed studies pooled prevalence rates without considering follow-up periods after symptoms [5].

According to the scientific literature, Long-COVID-19 syndrome is more prevalent in the working population. The evidence shows that the prevalence of symptoms was higher in people younger than 70 years old and, specifically, among 35–49-year-olds and 50–69-year-olds, compared to the general population five weeks after testing positive for COVID-19 [6,7].

In addition to the health problems associated with their condition, people in the working-age population with Long-COVID-19 syndrome also report adverse effects and stressful situations that affect their quality of life and occupational well-being [8]. Fear of job loss and future job insecurity, quarantine, unsafe work environments, infection and/or spreading the infection to those close to them for those working in “frontline” jobs, and COVID-19-related discrimination and/or stigma are all additional factors that may worsen the psychological state, creating a societal burden in this population [9,10].

Another relevant Long-COVID-19 symptom that can generate a significant burden on society is chronic pain [11]. Some authors [12,13] have reported pain as an important persistent symptom amongst COVID-19 survivors. Pain should be approached from the biopsychosocial model which understands this symptom as a complex, dynamic interaction of biological factors with psychosocial factors, which influences in a determinant way the coping strategies of pain. Consequently, these factors impact the chronification of pain, the development of disability, the appearance of a fear of movement, decreased activity levels and, therefore, decisive modification of the patient’s prognosis [14,15].

In a descriptive study of pain in survivors of COVID-19, the authors found a 19.6% prevalence of de novo chronic pain that interfered with their ability to function. Their pain was located primarily in the head and neck, but frequently occurred in the lower limbs, and it often moved around the body [16]. While the data and clinical experience suggest that pain is common in survivors of COVID-19, only a few studies have provided information on the pain experience of these patients, and none compared Long-COVID-19 patients’ de novo pain with successfully recovered COVID-19 patients and healthy controls. Therefore, the purposes of this study were to evaluate the health-related quality of life and characteristics of pain (i.e., pain intensity, pain interference, clinical presentation) in Long-COVID-19 patients and compare the location of pain with those who had successfully recovered from COVID-19 and healthy matched controls.

## 2. Materials and Methods

### 2.1. Study Design and Participants

A cross-sectional case-control study was performed. Using the study design and the recommended guidelines for the design of observational studies, the criteria and Strengthening the Reporting of Observational Studies in Epidemiology (STROBE) checklist were applied [17]. We conducted this study in accordance with the Declaration of Helsinki 1975, revised in 2013 [18]. Ethical approval for this study was obtained from the Biomedical Research Ethics Committee of Granada.

Three groups of patients were included in this study. The case group was composed of Long-COVID-19 patients meeting the WHO definition for this disease: “it is defined as the continuation or development of new symptoms 3 months after the initial SARS-CoV-2 infection, with these symptoms lasting for at least 2 months with no other explanation”. [19]. In addition, two additional groups of patients matched for age and gender were included: a group consisting of patients who successfully recovered from COVID-19 and, finally, a group of healthy controls who did not undergo SARS-CoV-2 infection. Patients in the Long-COVID-19 group were recruited from the autonomic Long-COVID-19 association. Control patients were recruited by word-of-mouth. Patients were recruited between May 2021 and April 2022.

Patients over 18 years of age who agreed to sign the informed consent form were included in the study. Patients were excluded if they had any of the following conditions: neurological or orthopedic pathologies that limited voluntary movement, a cognitive impairment that prevented them from understanding and answering the questionnaires, or if they had experienced reinfection with SARS-CoV-2. In addition, all patients who had been hospitalized due to COVID-19 infection and those who had pre-existing chronic pain according to the current IASP definition [20,21] before COVID-19 infection were excluded.

### 2.2. Outcome Measures

Patients were initially contacted by telephone to inform them of the study and to arrange a face-to-face assessment. Once informed consent was obtained, an assessment of demographic characteristics, pain characteristics, and clinical presentation of pain were performed.

Comorbidities were assessed by the Charlson comorbidity index, one of the most widely used scoring systems for assessing comorbidities that has been validated for use in several disorders [22]. In addition, the Charlson comorbidity index has been validated and previously used in the study of other respiratory pathologies, including patients infected by COVID-19 [23,24,25].

Pain intensity and interference were measured with the Brief Pain Inventory (BPI). The pain intensity section of the BPI is composed of four items and the pain interference section is composed of seven items. For the intensity section, the responses range from 0 (no pain) to 10 (worst pain) and for the interference section, the responses range from 0 (no interference) to 10 (total interference). To obtain the severity and interference index, the mean of the corresponding items is calculated, obtaining values between 0 to 10, with a higher score reflecting greater pain intensity and interference. The BPI has been established as a reliable and valid tool for assessing pain severity and interference [26,27] and it has previously been used and validated in other respiratory pathologies [28,29].

The Short-Form McGill Pain Questionnaire (SF-MPQ) is a version of the original McGill Pain Questionnaire [30] developed by Melzak in 1987 [31]. This scale is divided into several parts. The first part consists of a list of 15 adjectives, including 11 sensory and 4 affective descriptors of pain (e.g., terrible, throbbing, etc.) on a scale ranging from 0 (none) to 3 (severe), giving an overall score ranging from 0 to 45, as well as two scores from 0 to 33 for the sensory subscale and 0 to 12 for the affective subscale. In addition, it includes a VAS scale that assesses the patient’s pain in the last week. Finally, it includes a Present Pain Intensity Scale (PPI). The PPI is based on a single item measuring overall pain intensity. Patients are asked about their current level of pain on a 5-point Likert scale ranging from 0 (no pain) to 5 (unbearable). This scale has been shown to have excellent psychometric properties [31,32,33] and has previously been used in other respiratory pathologies [29,34].

The Widespread Pain Index (WPI) [35,36] assesses the bodily distribution of pain and specifically quantifies the degree of widespread body pain. The WPI assesses the presence of pain at 19 designated body sites over the past 7 days (e.g., neck, right lower arm, right upper leg). For each area with pain, the score is 1. The items are summed to give a total score ranging from 0 to 19 with higher scores indicating greater generalized pain. This questionnaire has previously been used and validated in other populations [37,38].

In addition, the same pain drawings used for the WPI test were digitized with a commercial scanner and imported into an image analysis program. The procedure used to digitize the pain drawings was previously described and had its reliability confirmed by Barbero et al. [39] This method of quantifying pain location and frequency is automated and does not rely on operator interpretation. This method allowed the generation of pain frequency and location maps that consisted of the overlay of all pain drawings, and these were analyzed in order to be able to illustrate where pain was most frequently perceived in the entire cohort. This was performed for both dorsal and ventral views. In this way, the pain drawings primarily provide us with information regarding pain frequency. The pain drawings of all participants were superimposed to illustrate where subjects reference pain most frequently, and for pain localization, the body graph was divided into regions and the percentage of participants referencing pain in specific, defined body regions is presented [40].

Health-related quality of life was assessed with the Euroqol-5 Dimensions 5 Levels Visual Analogue Scale (EQ-5D-5L) [41]. This is a widely used questionnaire consisting of 5 dimensions (“mobility”, “self-care”, “usual activities”, “pain or discomfort”, and “anxiety or depression”). Each of the dimensions has 5 possible levels. In addition, a visual analogue scale (VAS) with values ranging from 0 (worst imaginable state of health) to 100 (best imaginable state of health) is included to assess perceived health status. The EQ-5D-5L available value sets are accessible at https://euroqol.org/eq-5d-instruments/eq-5d-5l-about/valuation-standard-value-sets/ (accessed on 3 April 2022). This questionnaire has previously been used and validated in respiratory patients and COVID-19 patients [42,43,44].

### 2.3. Statistical Analysis

The statistical power calculation (GPower version 3.1.9.2 for Windows) was performed at the design stage based on our previous pilot study that employed a similar methodology (unpublished). This suggested that a sample size of 64 in each group would have 95% power to detect a probability of 0.5. To allow for a dropout rate of 10%, we decided to include approximately 71 patients in each study group.

Data were analyzed using the Statistical Package of Social Science (SPSS) program for Windows (version 26 IBM, Armonk, NY, USA). The normality of the data was first tested with the one-sample Kolmogorov–Smirnov test. For nominal variables, the chi-square test was used to identify differences. One-way analysis of variance (ANOVA) was used to compare the three groups when the continuous variables were normally distributed and Kruskal–Wallis was used when the continuous variables had a non-normal distribution. A 95% confidence interval was used for the statistical analysis. A *p*-value of ≤0.05 was set to indicate significant differences. No attempt at imputation was made for missing data.

## 3. Results

**Figure 1:**
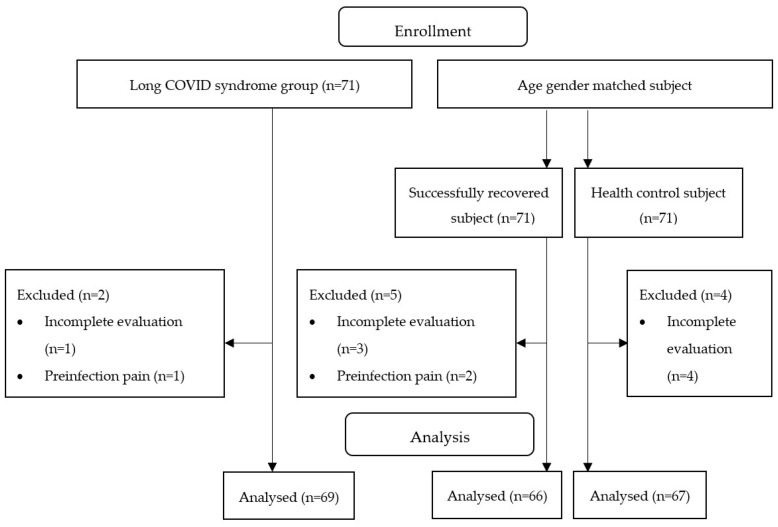
Flow diagram.

A total of 213 participants agreed to participate in this study and were considered eligible. The distribution of participants is shown in Figure 1.

The descriptive characteristics of the sample are shown in Table 1.

No statistically significant differences were found in the demographic characteristics of the participants. Statistically significant differences were found regarding the use of pharmacological pain treatment. In the Long-COVID-19 syndrome group, women showed a 20% higher drug intake. The drugs most commonly consumed by women were NSAIDs and metamizole. On the other hand, the drugs most consumed by men were NSAIDs and Paracetamol.

The pain characteristics are presented in Table 2.

Significant differences were found in pain intensity and pain interference between the Long-COVID-19 syndrome group, successfully recovered group, and healthy control group. However, no significant differences were found in pain intensity and pain interference between the successfully recovered group and healthy controls.

The SF-MPQ sensory and affective subscales also presented significant differences between groups, with poorer results in the Long-COVID-19 syndrome group. In addition, patients in the Long-COVID-19 syndrome group had greater pain in the last week compared to the group of successfully recovered patients and the healthy control group, measured with the VAS and, at the time of evaluation, the PPI.

Table 3 shows the health-related quality of life and widespread body pain results.

Statistically significant differences were found, with poorer results for the Long-COVID-19 group compared to the successfully recovered group and healthy control group for the EQ-5D subscales, as well as for the VAS scale. Regarding widespread body pain, the Long-COVID-19 group presented higher levels of widespread body pain, reaching statistically significant differences compared to the successfully recovered group and healthy control group.

**Figure 2:**
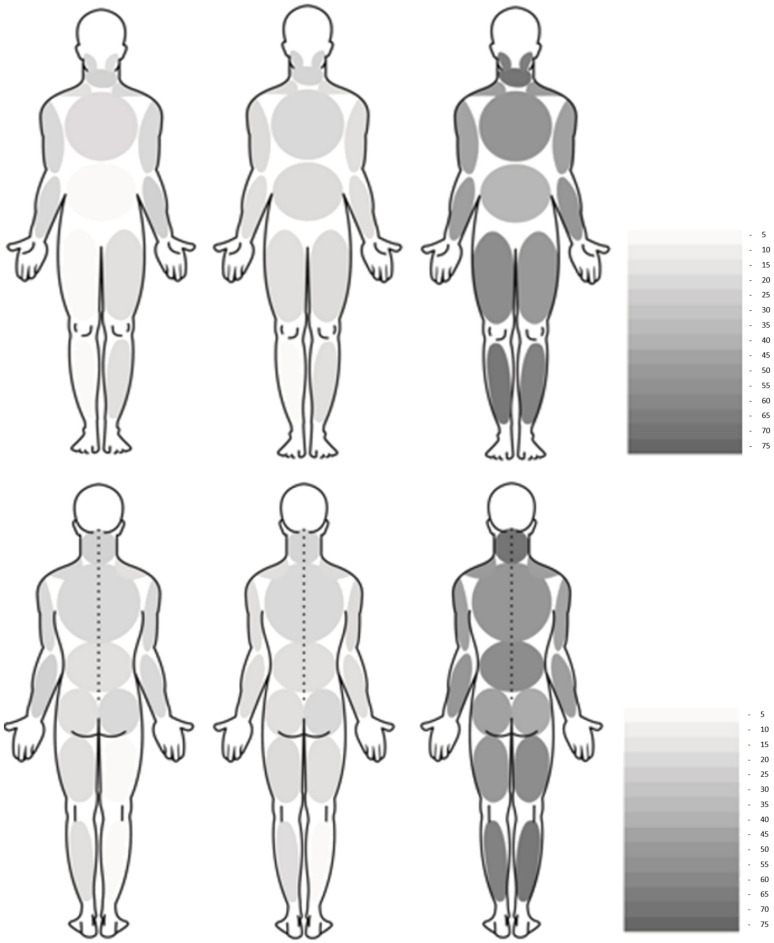
Pain drawings. Pain frequency maps were generated by superimposing the pain drawings of all patients included in the study. Pain frequency maps have been generated for both the dorsal and ventral views. The gray gradient indicates the percentage of people who reported pain in that specific area.

Figure 2 shows the location and frequency of pain. Specifically, it shows the percentage of patients presenting with pain in each area of the body associated with a grey scale, where the higher the percentage, the darker the color of that area. The number of patients presenting with pain in each area was much higher in the Long-COVID-19 group with the most frequent locations being the neck (69.1%), followed by the legs (68%) and the head (63.9%).

## 4. Discussion

This study aimed to evaluate the characteristics of pain (i.e., pain intensity, pain interference, clinical presentation) in Long-COVID-19 patients and compare the location of pain with those of successfully recovered COVID-19 patients and healthy matched controls. Our results show that patients with Long-COVID-19 syndrome have higher levels of pain intensity and interference, as well as greater pain generalization compared to the successfully recovered group and healthy control group. In addition, these patients show worse levels of health-related quality of life.

The sample of subjects included in this study was representative of the general population with Long-COVID-19 syndrome, demonstrating similar sociodemographic characteristics [45,46]. The higher prevalence of Long-COVID-19 syndrome in the female gender has previously been demonstrated. These differences in prevalence are generated because of different symptomatic, inflammatory, and immune responses between men and women [47,48,49]. Differences in immune system function between women and men may be a differential factor in terms of the development of Long-COVID-19 syndrome. Women’s ability to develop a more rapid and robust innate and adaptive immune response protects them from initial infection and prevents the risk of a greater severity of acute infection, unlike men who have a greater risk of more severe acute infection [50]. However, this quality that may protect them during the acute phase of infection may make females more vulnerable to prolonged autoimmune disease [51,52]. Another possible hypothesis to explain the sex differences is the hormonal difference, which may contribute to the asymmetry in risks and outcomes between genders, and the overlap of the symptoms of Long-COVID-19 syndrome with those of perimenopause and menopause may also have an influence [53].

With respect to differences in pharmacological treatment, although the pharmacological options tested so far to treat the different symptoms of COVID-19 have been numerous, they have demonstrated different levels of evidence in terms of efficacy and safety, with information on sex-related differences being limited. Sex-related differences in effectiveness are mainly explained by differences in the pharmacokinetic profile between men and women, as well as by sexual hormonal status [54]. Future research into the pharmacological treatment of COVID-19 should focus on generating adequate knowledge of gender and age as key factors of the individual variation in drug responses [55].

To date, the exact causes that generate pain in Long-COVID-19 patients have not yet been found. The scientific evidence published to date supports the fact that pain is a very common symptom in Long-COVID-19 patients, with a prevalence that varies greatly between studies depending on the target population, the time since acute infection, and the treatments received. The proposed mechanisms of the generation of this pain are an inflammatory response induced by the virus and prolonged in time, associated with the increase in pro-inflammatory cytokines and hyperactivation of immune system cells [45]. Another proposed mechanism for the generation of pain is the direct entry of the virus into nervous system cells and muscle cells mediated by angiotensin-converting enzyme 2 (ACE2) receptors. Finally, Fernández-de-las-Peñas and collaborators propose the hyperexcitability of the central and peripheral nervous system induced by the virus as the basis for pain with nociplastic characteristics, enhanced and prolonged in time by a series of negative psychosocial factors such as insomnia, stress, anxiety, and social isolation [56].

Although previous studies [57,58] have already reported a higher prevalence of pain in non-hospitalized patients than in hospitalized patients, so far, few studies have focused on studying this symptom in non-hospitalized patients [59,60,61,62,63,64]. These studies have focused on measuring the prevalence and main locations of pain without measuring pain characteristics. In addition, these studies have not excluded patients who had pain previously. Therefore, to our knowledge and to date, this is the first article that focuses on evaluating the characteristics of new-onset pain in non-hospitalized patients with Long-COVID-19 syndrome.

Many studies have demonstrated the presence of new-onset pain after infection with SARS-CoV-2, which can lead to chronic pain if not adequately studied and treated [64,65,66]. This new-onset pain has a multifactorial etiology, the basis of which is a prolonged proinflammatory state due to the immune system response to SARS-CoV-2 infection [67,68,69,70,71]. In addition to these physiological factors, we must add the alteration of the biopsychosocial factors [49,72,73] of patients due to the pandemic situation, resulting in a new pattern of pain in these patients that needs to be defined and characterized [66].

The proportion of patients with Long-COVID-19 syndrome presenting with new-onset pain in this study was 69.5%, which is quite high when compared with the prevalence of chronic pain in other populations with respiratory diseases; for example, in patients with COPD, the prevalence of chronic pain ranges from 32–62% [29,74,75]. Furthermore, the proportion of patients presenting with new-onset chronic pain in our study was also much higher than in previous studies of patients who had undergone COVID-19. For example, in a study by Soares et al., the prevalence of pain in COVID patients was 19.6% [16]. This may be because the patients included in the study were already diagnosed with Long-COVID-19 syndrome in contrast to previous studies [16,44].

However, other studies previously performed at different clinical time points of SARS-CoV-2 infection already showed a similar prevalence of pain [32,52,53]. For example, studies performed during the acute phase of the disease, such as the study by Mural et al. [76] and Oguz-Akarsu et al. [77] showed a proportion of pain in their sample of 69.3% and 71.6%, respectively.

The results obtained from this study show a generalized pain pattern, with the most frequent location of pain being the neck, followed by the legs and head. These results do not agree with the results shown by other studies where the most frequent location of pain is the back [78,79,80]. Concerning the generalized pain pattern, our results are consistent with previous [11,81] studies, but not with other studies showing a more localized pain pattern [16,63,82,83]. Concerning headaches, this study shows a higher prevalence than studies previously conducted in patients who had been hospitalized [84,85,86,87,88,89]. Concretely, our results show that 63.9% of patients reported headaches, while the study by Fernández-de-las-Peñas et al. [84] and the study by González-Martínez et al. [89] reported 23.4% and 13%, respectively.

Although the exact mechanisms that generate pain in patients with Long-COVID syndrome at different body sites have not yet been elucidated, there are different theories that attempt to explain it. Regarding head and neck pain, it is believed that it may be a direct consequence of complications generated by the viral infection such as hypoxia, dehydration, and fever [90]. In addition, findings of increased IL-10 levels in COVID-19 patients presenting with headaches seem to indicate that headaches may be a consequence of high cytokine levels [91,92]. Finally, another hypothesis for the cause of headaches in these patients is the ability of SARS-CoV-2 to invade the central nervous system [93,94]. Pain at the lower limb level could actually be explained by joint pains or peripheral neuropathies, but these hypotheses remain theoretical so far [95,96].

The mean pain intensity of the Long-COVID-19 syndrome patients included in this study measured with the BPI scale is 5.12. These results are in line with previously conducted studies showing moderate pain intensity [49,97,98]. The study by Soares et al. [16] in which new-onset pain was evaluated showed levels of intensity and interference higher than those shown in our results, as well as a pain location similar to that of this study. However, we must consider that this study was performed on patients who had been hospitalized.

The results of this study demonstrate a lower health-related quality of life in Long-COVID-19 syndrome patients compared to the successfully recovered group and healthy control group. These results are in line with previously conducted research which demonstrated that patients with COVID-19 [86] and Long-COVID-19 syndrome [11,99,100,101,102] have worse levels of health-related quality of life.

The patient’s perception of the disease are frameworks that patients construct in order to make sense of their symptoms and medical conditions. Thus, the patient’s behavior and control of the disease will depend on the patient’s cognitive representation of the disease. This cognitive representation of the disease will, in turn, be influenced by beliefs about the disease and what it means for the patient’s life. The main factors that influence the patient’s perception of the disease are the symptoms that form it, the control that the patient has over these symptoms, previous personal and family experiences, and the consequences that the disease generates in the life of the patient and his or her family. Numerous publications show that the perception of the disease is related to important health outcomes such as functionality and perceived state of health, and these can have a relevant influence on their measurement [103,104]. Thus, patients who have undergone an acute disease process in recent months may have a completely altered perception of the disease, leading to a more optimistic view of their health-related quality of life once they have recovered [105]. This would justify the reason why patients who have had COVID-19 may have a better health-related quality of life than those who have never had the disease.

The differences in our results from previously conducted studies may be due to several factors. First, many of the studies performed so far have had a positive antigen or polymerase chain reaction (PCR) test as an inclusion criterion. In our study, following the WHO definition of Long-COVID-19 syndrome [19], patients with a probable or confirmed history of SARS-CoV-2 infection were included. In addition, we must take into account the differences in the patient sample in terms of time since infection, which was longer than in other studies, hospitalization, since only non-hospitalized patients were included, and new-onset chronic pain, since only patients without previous pain were included.

Several limitations of this study should be noted. First, it had a small sample size; larger sample sizes could improve the reliability of the results. However, previous studies in this population have used similar sample sizes [11,16]. Another limitation of this study is the use of tests based on Classical Test Theory instead of Item Response Theory, which has less bias in the scores and better psychometric properties [106,107]. As another limitation of this study, we could include the fact that the sample was obtained from an association of patients with Long-COVID-19 syndrome, which makes the patients included more proactive in seeking help, perhaps because they have a greater intensity of symptoms. Despite having described several characteristics of new-onset chronic pain in patients with Long-COVID-19 syndrome, there are still important pain-related aspects, such as a more comprehensive assessment of patients’ mood. It would also be interesting to include an evaluation of serum biomarkers in patients with Long-COVID-19 syndrome to assess changes in these and compare them with the successfully recovered group and healthy control group. In addition, a longitudinal design would allow us to observe changes in pain levels over time. Another limitation of this study is the use of scales that have not been specifically validated in the study population.

## 5. Conclusions

In conclusion, compared with healthy controls and patients who successfully recovered from COVID-19, the results of this study show that health-related quality of life is significantly worse, and pain is significantly more prevalent in patients with Long-COVID-19 syndrome. The pain of these patients is characterized by widespread pain of moderate intensity and interference, with the most frequent locations being the neck, legs, and head, and this pain significantly effects the quality of life of these patients. These results will help inform the design of programs tailored to the needs of this population.

## Figures and Tables

**Table 1:** Descriptive characteristics of the sample.

Variables	Long-COVID-19 Syndrome Group (n = 69)	Successfully Recovered COVID-19 Group (n = 66)	Healthy Control Group (n = 67)	F
Age	44.99 ± 2.79	44.90 ± 2.94	44.67 ± 3.11	0.144
Sex (% female)	75.26	75.79	75.51	0.973
Weeks since infection	104.23 ± 11.36	103.26 ± 8.96		0.300
BMI (kg/m^2^)	25.16 ± 3.07	25.19 ± 3.19	25.38 ± 3.6	0.052
Smoker (%)	13.0	22.9	17.2	0.432
Other diseases (%)	31.5	22.9	24.1	0.497
Charlson index	0.17 ± 0.38	0.15 ± 0.41	0.24 ± 0.51	0.488
Non-pharmacological treatment (%)	39.2	24.6	20	0.056
Physiotherapy	76.32	64.29	83.33	
Psychology	23.68	35.71	16.66	
Pharmacological treatment for pain (%)	74.2	14	13.3	<0.001 **
NSAIDs	31.9	50	25	
Paracetamol	19.4	37.5	50	
Muscle relaxants	13.9	12.5	25	
Tramadol	12.5	0	0	
Codeine	2.8	0	0	
Metamizole	19.4	0	0	

BMI: Body Mass Index; NSAIDs: Nonsteroidal Anti-inflammatory Drugs. Descriptive data for each group are expressed as means ± standard deviation and percentages when appropriate. ** *p* < 0.001.

**Table 2:** Pain characteristics.

Variables	Long-COVID-19 Syndrome Group (n = 69)	Successfully Recovered COVID-19 Group (n = 66)	Healthy Control Group (n = 67)	F
Pain prevalence (%)	69.5	26.3	23.3	<0.001 *
BPI-intensity	5.12 ± 2.28	0.93 ± 1.74	0.75 ± 1.48	101.88 ^bc^
BPI-interference	5.78 ± 2.77	0.82 ± 1.87	0.51 ± 1.41	107.73 ^bc^
SF-MPQ-sensory subscale	15.99 ± 6.89	2.94 ± 4.43	2.8 ± 5.54	108.79 ^bc^
SF-MPQ-affective subscale	5.75 ± 3.33	0.83 ± 1.29	0.67 ± 1.84	81.54 ^bc^
SF-MPQ-overall score	21.74 ± 9.51	3.77 ± 5.62	3.47 ± 6.96	114.46 ^bc^
SF-MPQ-VAS	65.11 ± 22.8	15.75 ± 22.92	11.62 ± 21.14	115.41 ^bc^
SF-MPQ-PPI	2.53 ± 1.35	0.49 ± 0.89	0.31 ± 0.66	78.36 ^bc^

BPI: Brief Pain Inventory; SF-MPQ: Short-Form McGill Pain Questionnaire; VAS: Visual Analogue Scale; PPI: Present Pain Intensity Scale. Descriptive data for each group are expressed as means ± standard deviation and percentages where appropriate. * *p* < 0.05. ^b^ = Significant differences between the healthy control group and Long-COVID-19 syndrome group. ^c^ = Significant differences between the successfully recovered group and Long-COVID-19 syndrome group.

**Table 3:** Health-related quality of life.

Variables	Long-COVID-19 Syndrome Group (n = 69)	Successfully Recovered COVID-19 Group (n = 66)	Healthy Control Group (n = 67)	F
WPI	10.02 ± 5.93	1.16 ± 2.52	0.9 ± 1.68	86.297 ^bc^
EQ-5D-mobility	2.36 ± 1.07	1.00 ± 0.00	1.17 ± 0.46	65.61 ^bc^
EQ-5D-self-care	1.80 ± 0.98	1.00 ± 0.00	1.03 ± 0.18	31.82 ^bc^
EQ-5D-usual activities	3.24 ± 0.95	1.04 ± 0.29	1.14 ± 0.44	225.40 ^bc^
EQ-5D-anxiety or depression	3.43 ± 0.76	1.26 ± 0.53	1.48 ± 0.68	216.35 ^bc^
EQ-5D-pain or discomfort	2.20 ± 1.17	1.47 ± 0.62	1.34 ± 0.61	18.98 ^bc^
EQ-5D VAS	42.05 ± 21.67	74.62 ± 28.31	70.76 ± 31.70	31.49 ^bc^

WPI: Widespread Pain Index; EQ-5D: Euroqol-5 dimensions; VAS: Visual Analogue Scale. Descriptive data for each group are expressed as means ± standard deviation and percentages where appropriate. ^b^ = Significant differences between the healthy control group and Long-COVID-19 group. ^c^ = Significant differences between the successfully recovered group and Long-COVID-19 group.

## Data Availability

No additional data are available.
